# Psychiatric illness, emotional distress, glycemic control and chronic complications in type 1 diabetes subjects

**DOI:** 10.20945/2359-3997000000386

**Published:** 2021-07-16

**Authors:** Thiago Malaquias Fritzen, Letícia Schwerz Weinert, Isabele Beatris Denk, João Alberto Succolotti Deuschle, Isabel Conte, Maurício Picolo Menegolla, Ticiana da Costa Rodrigues

**Affiliations:** 1 Universidade Federal do Rio Grande do Sul Programa de Endocrinologia Porto Alegre RS Brasil Programa de Endocrinologia, Universidade Federal do Rio Grande do Sul (UFRGS), Porto Alegre, RS, Brasil.; 2 Universidade Federal de Pelotas Hospital Escola Divisão de Endocrinologia Pelotas RS Brasil Divisão de Endocrinologia, Hospital Escola, Universidade Federal de Pelotas (UFPEL), Pelotas, RS, Brasil.; 3 Universidade Federal de Pelotas (UFPEL) Pelotas RS Brasil Universidade Federal de Pelotas (UFPEL), Pelotas, RS, Brasil.; 4 Universidade Federal do Rio Grande do Sul Porto Alegre RS Brasil Universidade Federal do Rio Grande do Sul (UFRGS), Porto Alegre, RS, Brasil.; 5 Hospital de Clínicas de Porto Alegre Divisão de Endocrinologia Porto Alegre RS Brasil Divisão de Endocrinologia, Hospital de Clínicas de Porto Alegre (HCPA), Porto Alegre, RS, Brasil.

**Keywords:** Type 1 diabetes, psychiatric disorders, anxiety, depression, diabetes complication

## Abstract

**Objectives:**

To assess the prevalence of psychiatric disorders in patients with type 1 diabetes mellitus (T1D) and to compare patients with and without psychiatric disorder.

**Materials and methods:**

We made a cross-sectional study including patients with T1D assisted in the outpatient clinics of the Brazilian National Health System. To assess depression and anxiety, we used the PHQ-9 questionnaire and the DSM-5th edition criteria, respectively. B-PAID evaluated the level of emotional distress associated with diabetes; EAT-26, eating disorders; SCI-R, adherence to the proposed clinical treatment.

**Results:**

We analyzed 166 patients aged 33 (22-45.2) years, 53.6% female. The prevalence of depression and anxiety was 20.5% and 40.4%, respectively. HbA1c was worse in the depressed (9.0% vs. 8.4%, p = 0.008), in the anxious ones (9.0% vs. 8.3%, p = 0.012) and in the patients with high levels of B-PAID (8.8 % vs. 8.3 %, p = 0.009). There was no difference in the prevalence of complications related to diabetes.

**Conclusions:**

The prevalence of psychiatric disorders and emotional distress related to diabetes was high in our population of T1D patients, and depression and high levels of B-PAID were associated with the worse glycemic control.

## INTRODUCTION

Type 1 diabetes mellitus (T1D) is a chronic autoimmune disease that causes absolute deficiency in insulin production. People with the disease need daily insulin doses and glycemic self-monitoring several times a day, in addition to healthy living habits, with a routine of diet and physical activity. This demand for continuous self-care is especially difficult in childhood and adolescence. The transition to adulthood, in addition to impacting psychological maturity and increasing independence, commonly leads to worsening of the metabolic control of diabetes (1), increasing the prevalence of psychiatric disorders in this transition phase (2).

According to WHO data, depressive disorder affects about 4.4% of the world population, while the estimated prevalence of anxiety is 3.6% (3). Suicide accounts for 1.5% of all deaths, being among the 20 main causes of general mortality (3). Among patients with diabetes, several studies show higher rates of depression and anxiety than in the general population (4–6). Diabetes distress, a specific emotional disorder related to the underlying disease (7,8), has an estimated prevalence of 18% to 45%, depending on the geographic population evaluated (9), being the most common psychological disorder. Approximately 20% of men and 30% of women with T1D have an eating disorder (10), which is associated with poor glycemic control and an increased risk of developing diabetes complications (11–13). Likewise, the risk of suicide is higher in the diabetic population compared to controls. Rates of 9% suicidal ideation and 16% suicide attempts were reported in a cohort of patients with T1D (14).

The aim of this study was to evaluate the prevalence of psychiatric disorders in patients with T1D and its relationship with adherence to treatment, emotional distress related to diabetes control, metabolic rate and increased risk of related complications in the short and long term in a cohort of patients in southern Brazil.

## MATERIALS AND METHODS

### Study design and population

Cross-sectional study carried out in specialized care clinics at two university centers in the cities of Pelotas and Porto Alegre, southern Brazil – Federal University of Pelotas and Federal University of Rio Grande do Sul. Patients with T1D who had performed more than 2 consultations registered in medical records, the last service being performed in the previous 12 months. The recruitment of patients took place from March 2016 to October 2019.

Patients under 10 years of age, those with other types of diabetes, those who had no registered consultation in the last 12 months or who did not agree to participate in the study were excluded.

The diagnosis of T1D was made by a specialist doctor using clinical and laboratory criteria, such as a history of diabetic ketoacidosis, exclusive treatment with insulin, presence of positive glutamic acid anti-decarboxylase antibody or C peptide below the reference value.

The research project was submitted to and approved by the ethics committees of the Medical School of the Federal University of Pelotas (approval number 2.994.677) and the *Hospital de Clínicas de Porto Alegre* (approval number 2.762.272). CAAE number 91767018.9.1001.5327.

All participants signed an informed consent form.

### Covariables and questionnaires

The variables under study were collected by previously trained interviewers, and data collection took place in three phases: 1. application of a face-to-face questionnaire to obtain socio demographic data, 2. three self-administered questionnaires previously standardized and translated into Portuguese (15–17) addressing aspects of the patient's relationship with diabetes and their current emotional situation; and 3. review of medical records to obtain clinical and laboratory data. The sampling was for convenience and consecutive, as scheduled for consultations. Participants from the Porto Alegre group were interviewed in a single approach, while those from Pelotas were interviewed in 2 different meetings, simultaneous to the consultations.

The clinical information obtained from the medical record was the presence of macrovascular disease (acute myocardial infarction [AMI], peripheral arterial obstructive disease or stroke), systemic arterial hypertension, dyslipidemia, kidney disease, diabetic neuropathy and retinopathy and hypoglycemia.

The laboratory tests evaluated in medical records within the last 12 months, were: glycated hemoglobin, the most recent (HbA1c, High performance liquid chromatography, HPLC), urine sample albuminuria (immunoturbidimetry), serum creatinine (colorimetric method), thyroid function (immunoassay of microparticles by chemiluminescence), antibodies to celiac disease (chemiluminescence method), anti-glutamic acid decarboxylase antibody (anti GAD, enzyme immunoassay method) and C peptide (microparticle chemiluminescent immunoassay).

Those ones with a medical record of the event were considered with macrovascular disease. Patients admitted as hypertensive and dyslipidemic were using antihypertensive and hypolipidemic medication, respectively. Participants who had albuminuria in isolated urine sample > 30 mg/g creatinine and/or estimated glomerular filtration (eGFR) < 60 mL/min/1.73 m² (Chronic Kidney Disease Epidemiology Collaboration, CKD-EPI) in two different times were considered renal patients (18,19). The diagnosis of diabetic neuropathy and diabetic retinopathy were obtained through medical records, since Semmes-Weinstein 10 g monofilament neuropathy test and the fundus examination of the eye are performed annually. Glycemia events ≤ 70 mg/dL were considered hypoglycemia (20), severe when needed the help of third parties for management; frequent when 3 or more weekly and night events occurred was related to 1 or more weekly episodes obtained from clinical information directly from the patient. Those patients who reported practicing at least 150 minutes of weekly physically activity were considered physically active.

Participants were asked about their previous diagnosis of psychiatric disorder indicating which disease(s) they had: depression, anxiety, panic disorder, bipolar disorder, “I don't know” – when they didn't know their psychiatric diagnosis – and “other”, when the pathology was not included in the presented relation. For objective evaluation, the Brazilian Problem Areas In Diabetes Scale (B-PAID) questionnaires, related to the level of emotional distress associated with the routine of living with diabetes, with values ≥ 40 indicating high level of emotional distress, considered “Severe B-PAID” (15). Eating Attitudes Test-26 (EAT-26), to access eating disorders (21) and Self Care Inventory – Revised Version (SCI-R) to estimate the degree of adherence to the proposed clinical treatment (17). The three questionnaires are validated for Portuguese (15–17). A participant with a score ≥ 10 on the Patient Health Questionnaire – 9 (PHQ-9) (22), validated for the Brazilian population (23), was considered to have a depressive disorder. The screening for anxiety was performed using the DSM-5th edition criteria (24). The two last one's screenings were used in this article to classify depressive and anxious patients. Participants were asked about the monthly family income, in minimum wages (the brazilian minimum wage is equivalent to US$ 200 dollars). Regarding drug use (licit or illicit) participants were considered users who self-reported routine use, without quantification. Patients were approached about ideation and/or attempted suicide through a direct question: “Have you ever had suicidal ideation or attempted suicide?”.

### Statistical analysis

For statistical analysis and graphic representation, we used the program Statistical Package for the Social Sciences (SPSS, IBM Corp. and its licensors) for Windows (version 22). Dichotomous variables were described as number and percentage, and quantitative variables were defined as mean, standard deviation, median and interquartile range according to the Gaussian distribution. Comparisons were made using t test for variables with normal distribution, and Mann-Whitney test for non-Gaussian distribution for continuous variables, and chi-square and Fisher's exact for qualitative variables. Logistic and linear regression models were used to assess possible effects of other additional risk factors. The level of significance adopted was 5%.

### Sample calculation

The sample calculation was based on a Brazilian study with T1D patients, whose prevalence of depression was 13.6% with a sample size of 181 patients being defined, assuming 5% as the maximum difference between the prevalence of real depression and estimated, with a 95% confidence level.

## RESULTS

Altogether, 191 patients met the inclusion criteria, with 25 being excluded for not completing the clinical interview. Among the participants, 120 came from the Porto Alegre outpatient clinic and 46 from Pelotas. Regarding the patients excluded from the study (n = 25), they were younger with a median of 21 years (15.0-27.5), had shorter disease duration and a higher level of HbA1c 9.4% [7.5%-12.1%], (79 mmol/mol [58-109]), than the patients included in the study. The other variables were not different. (Supplement).

The analysis of the 166 patients included showed that 89 (53.6%) were women, with a median of 14 years of disease duration, HbA1c median of 8.5% (7.8%-9.4%), (69 mmol/mol [62-79]), and monthly family income of 2 (1–3) minimum wages (Supplement). All those included used a basal/bolus insulin regimen, with 91 (54.8%) using NPH (Neutral Protamine Hagedorn) insulin, while the others used insulin glargine, n = 75 (45.2%), as basal therapy. Of the prandial insulins, 111 (66.9%) used rapid-acting insulin analogues (lispro, aspart or glulisine) and the remaining patients used regular human insulin. Only one patient was an intermittent insulin pump user. Of those who used a basal/bolus regimen, 119 (71.7%) made 2 to 4 applications a day, with 136 (81.9%) reusing needles and/or syringes three or more times. Almost all patients checked capillary blood glucose daily, n = 156 (94%), with 137 (82.5%) taking 3 or more tests a day. Only 18 (10.8%) patients had excellent glycemic control (HbA1c ≤ 7.0% or 53 mmol/mol) (9).

**Figure 1:**
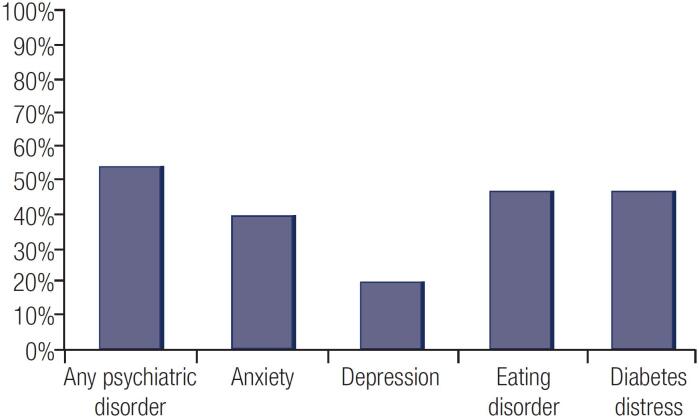
Prevalence of Psychiatric Disorders in Patients with T1D.

The prevalence of some psychiatric disorder in the sample studied was n = 91 (54.8%), with 67 (40.4%) patients having positive screening for anxiety and 34 (20.5%) for depressive disorder, exclusively (Figure 1). Of the 91 patients with positive screening, 62 (68.1%) had already been evaluated by psychologists/psychiatrists at some point in their lives. Psychiatric drug users were 45 (27.1%), of 166 participants.

A comparative analysis was performed between the groups of patients with and without depression assessed by PHQ-9, which are described in Table 1. Glycemic control, assessed by measuring HbA1c, was worse in depressed patients (9.0% vs. 8.45%, p = 0.008), (75 mmol/mol vs. 69 mmol/mol), requiring greater family support (23 [67.6%] vs. 63 [47.7%], p = 0.038) in relation to controls. No statistically significant differences were found in acute and chronic complications related to diabetes in relation to the presence of depression, although depressed patients were more prone to severe hypoglycemia (23 [67.6%] vs. 74 [56.1%], p = 0.22) and had greater need for hospitalization due to the diabetes (22 [64.7%] vs. 75 [56.8%], p = 0.405). We assessed, through correlation, possible factors associated with glycemic control and observed only a weak correlation with income (r = 0.206, p = 0.002). The other factors were not correlated. In the multiple regression analysis using HbA1c as the main outcome and adjustment for diabetes time, we found that the presence of depression was associated with the worse glycemic control (β = 0.202; p = 0.013, CI 95% 0.177-1.485).

**Table 1:** Demographic characteristics of type 1 diabetic patients with or without depression (n = 166)

Variable			Depression (n = 34)	Without Depression (n = 132)	p
Age, years			34 (28-45.5)	33 (21-45.5)	0.327
Age of diagnosis, years			14.5 (11.7-27.2)	18 (11-25)	0.773
Years with T1D			18 (7.7-25.2)	12 (5-25)	0.176
Body mass index			24.9 ± 4.6	24.9 ± 4.1	0.694
Glycated hemoglobin		%	9 (8.4-10.3)	8.4 (7.7-9.2)	**0.008**
		mmol/mol	74.8 (68.3-89.1)	68.3(60.6-77)	
Gender					
	Female		21 (61.8)	68 (51.5)	0.285
Skin Color					0.226
	White		26 (76.5)	116 (87.9)	
	Black		5 (14.7)	11 (8.3)	
	Brown		3 (8.8)	5 (3.8)	
Nephropathy			10 (29.4)	28 (21.4)	0.321
Neuropathy			8 (23.5)	16 (12.1)	0.092
Vasculopathy			4 (11.8)	7 (5.3)	0.177
Diabetic retinopathy			10 (29.4)	44 (33.3)	0.789
Dyslipidemia			10 (29.4)	25 (18.9)	0.182
Hypertension			11 (32.4)	40 (30.3)	0.860
Hypoglycemia					
	Severe		23 (67.6)	74 (56.1)	0.222
	Frequent		11 (32.4)	27 (20.5)	0.141
	Nocturnal		18 (52.9)	50 (37.9)	0.111
Previous ICU admission			13 (38.2)	58 (43.9)	0.549
Diabetes hospitalization			22 (64.7)	75 (56.8)	0.405
Family support			23 (67.6)	63 (47.7)	**0.038**
Physical activity			10 (29.4)	58 (43.9)	0.125
Glucose self-monitoring			31 (91.2)	125 (94.7)	0.442
Psychiatric medication			19 (55.9)	26 (19.7)	**<0.001**
Psychological assessment			30 (88.2)	59 (44.7)	**<0.001**
Drugs					
	Alcohol		3 (8.8)	27 (20.5)	0.116
	Cigarette		4 (11.8)	12 (9.1)	0.638
	Cocaine		2 (5.9)		**0.005**
Suicidal			18 (52.9)	18 (13.6)	**<0.001**
EAT ≥ 21			21 (61.8)	58 (43.9)	0.063
SCI-R > 48			14 (41.2)	77 (58.3)	0.073
B-PAID, punctuation			60.6 (39.3-71.2)	35 (14.1-55)	**<0.001**
Anxiety			23 (67.6)	44 (33.3)	**<0.001**

Data are presented as median (interquartile range), means ± standard deviation and number of patients (percentual). Psychiatric medication means current treatment. Psychological assessment corresponds to assessment at any time in the past.

The comparison between groups of patients with and without anxiety (Table 2), on the other hand, also demonstrated that anxious patients have the worse glycemic control (9.0% [8.3%-9.6%] vs. 8.3% [7.8%-9.1%], p = 0.012), (75 mmol/mol [67-81] vs. 67 mmol/mol [62-76]). In addition, anxiety was more frequent in females (45 [67.2%] vs. 44 [44.4%], p = 0.004), in participants with ideation or suicide attempts (26 [38.8%] vs. 10 [10.1%]), p < 0.001) and it was lower in physical activity practitioners (18 [26.9%] vs. 50 [50.5%], p = 0.002) compared to patients without anxiety. The other variables showed no significant difference. After regression adjusted for sex, physical activity and diabetes duration, anxiety was not associated with the worse glycemic control.

**Table 2:** Demographic characteristics of type 1 diabetic patients with or without anxiety (n = 166)

Variable			Anxiety (n = 67)	Without Anxiety (n = 99)	p
Age, years			33 (24-47)	33 (19-45)	0.507
Age of diagnosis, years			17 (12-25)	16 (11-26)	0.557
Years with T1D			15 (6-25)	14 (6-25)	0.699
Body mass index			24.8 ± 4.2	25 ± 4.3	0.682
Glycated hemoglobin		%	9.0 (8.3-9.6)	8.3 (7.8-9.1)	**0.012**
		mmol/mol	74.8 (67.2-81.4)	67.2 (61.7-75.9)	
Gender					
	Female		45 (67.2)	44 (44.4)	**0.004**
Skin Color					0.394
	White		55 (82.1)	87 (87.9)	
	Black		9 (13.4)	7 (7.1)	
	Brown		3 (4.5)	5 (5.1)	
Nephropathy			20 (29.9)	18 (18.4)	0.085
Neuropathy			13 (19.4)	11 (11.1)	0.136
Vasculopathy			6 (9)	5 (5.1)	0.321
Diabetic retinopathy			23 (34.3)	31 (31.3)	0.666
Dyslipidemia			12 (17.9)	23 (23.2)	0.41
Hypertension			20 (29.9)	31 (31.3)	0.471
Hypoglycemia					
	Severe		44 (65.7)	53 (53.5)	0.12
	Frequent		18 (26.9)	20 (20.2)	0.316
	Nocturnal		29 (43.3)	39 (39.4)	0.617
Previous ICU admission			31 (46.3)	40 (40.4)	0.454
Diabetes hospitalization			40 (59.7)	57 (57.6)	0.785
Family support			40 (59.7)	46 (46.5)	0.094
Physical activity			18 (26.9)	50 (50.5)	**0.002**
Glucose self-monitoring			60 (89.6)	96 (97)	**0.049**
Psychological assessment			43 (64.2)	46 (46.5)	**0.025**
Psychiatric medication			25 (37.3)	20 (20.2)	**0.015**
Drugs					
	Alcohol		12 (17.9)	18 (18.2)	0.964
	Cigarette		7 (10.4)	9 (9.1)	0.771
	Cocaine		2 (3)		0.084
Suicidal			26 (38.8)	10 (10.1)	**<0.001**
EAT ≥ 21			34 (50.7)	45 (45.5)	0.503
SCI-R > 48			31 (46.3)	60 (60.6)	0.069
B-PAID, punctuation			56.2 (37.5-70)	25 (11.2-47.5)	**<0.001**
Depression			23 (34.3)	11 (11.1)	**<0.001**

Data are presented as median (interquartile range), means ± standard deviation and number of patients (percentual). Psychiatric medication means current treatment. Psychological assessment corresponds to assessment at any time in the past.

As for the eating disorder, 79 (47.6%) patients had positive EAT screening. The group of patients with positive screening had an older age, were more frequently female, had a higher rate of clinical comorbidities and a higher level of suffering from B-PAID (Table 3).

**Table 3:** Demographic characteristics of type 1 diabetic patients at EAT Questionnaire^1^ (n = 166)

Variable			Eating disorder (n = 79)	Negative eating disorder (n = 87)	p
Age, years			37 (24-50)	31 (21-39)	**0.022**
Age of diagnosis, years			18 (11-26)	16 (11-25)	0.46
Years with T1D			18 (6-27)	11 (6-20)	0.078
Body mass index			25.6 ± 4.5	24.3 ± 3.9	0.072
Glycated hemoglobin		%	8.7 (7.9-9.8)	8.5 (7.6-9.1)	0.068
		mmol/mol	71.6 (62.8-83.6)	69.4 (59.5-76)	
Gender					
	Female		51 (64.6)	38 (43.7)	**0.007**
Skin color					0.501
	White		65 (82.3)	77 (88.5)	
	Black		9 (11.4)	7 (8)	
	Brown		5 (6.3)	3 (3.4)	
Nephropathy			57 (72.2)	70 (81.4)	0.159
Neuropathy			15 (19)	9 (10.3)	0.114
Vasculopathy			7 (8.9)	4 (4.6)	0.27
Diabetic retinopathy			34 (43)	20 (23.0)	**0.011**
Dyslipidemia			22 (27.8)	13 (14.9)	**0.042**
Hypertension			33 (41.8)	18 (20.7)	**0.006**
Hypoglycemia					
	Severe		48 (60.8)	49 (56.3)	0.562
	Frequent		18 (22.8)	20 (23)	0.975
	Nocturnal		38 (48.1)	30 (34.5)	0.075
Previous ICU admission			36 (45.6)	35 (40.2)	0.487
Diabetes hospitalization			46 (58.2)	51 (58.6)	0.959
Family support			41 (51.9)	45 (51.7)	0.982
Physical activity			36 (52.9)	32 (47.1)	0.25
Glucose self-monitoring			73 (92.4)	83 (95.4)	0.418
Psychological assessment			51 (64.6)	38 (43.3)	**0.007**
Psychiatric medication			29 (36.7)	16 (18.4)	**0.008**
Drugs					
	Alcohol		11 (13.9)	19 (21.8)	0.186
	Cigarette		5 (6.3)	11 (12.6)	0.169
	Cocaine		1 (1.3)	1 (1.1)	0.945
Suicidal			21 (26.6)	15 (17.2)	0.145
SCI-R >48			50 (63.3)	41 (47.1)	**0.037**
B-PAID, punctuation			42.5 (27.5-65)	35 (11.2-57.5)	**<0.001**
Depression			21 (26.6)	13 (14.9)	0.063
Anxiety			34 (43)	33 (37.9)	0.503

Data are presented as median (interquartile range), means ± standard deviation and number of patients (percentual). Psychiatric medication means current treatment. Psychological assessment corresponds to assessment at any time in the past.

1 Eating Attitudes Test - 26. values ≥ 21 mean that patient is at risk of eating disorders.

The B-PAID instrument diagnosed 79 (47.6%) patients with high emotional load, with glycemic control being worse in this group. The group with the greatest emotional distress had a higher percentage of women and more patients with positive screening for eating disorder. The individuals most adherent to the treatment were in the group with the lowest emotional distress and those who practiced regular physical activity had significantly lower levels of emotional distress (Table 4). B-PAID maintained an association with worse glycemic control after adjusting for sex and regular physical activity after multiple regression analysis (β = 0.2; p = 0.018, CI 95% 0.117-1.212).

**Table 4:** Demographic Characteristics of Type 1 Diabetic Patients at B-PAID^1^ Questionnaire (n = 166)

Variable			Severe B-PAID 2 (n = 79)	Mild B-PAID (n = 87)	p
Age, years			32 (24-45)	34 (21-47)	0.997
Age of diagnosis, years			18 (12-25)	16 (11-26)	0.231
Years with T1D			11 (6-23)	16 (6-27)	0.276
Body mass index			25.3 ± 4.46	24.6 ± 4.01	0.286
Glycated hemoglobin		%	8.8 (7.9-10.1)	8.3 (7.6-9.0)	**0.009**
		mmol/mol	72.7 (62.8-86.9)	67.2 (59.5-74.9)	
Gender					
	Female		53 (67.1)	36 (41.4)	**<0.001**
Skin Color					0.442
	White		65 (82.3)	77 (88.5)	
	Black		10 (12.7)	6 (6.9)	
	Brown		4 (5.1)	4 (4.6)	
Nephropathy			19 (24.1)	19 (22.1)	0.765
Neuropathy			14 (17.7)	10 (11.5)	0.255
Vasculopathy			5 (6.3)	6 (6.9)	0.883
Diabetic retinopathy			24 (30.4)	30 (34.5)	0.524
Dyslipidemia			14 (17.7)	21 (24.1)	0.311
Hypertension			20 (25.3)	31 (35.6)	0.22
Hypoglycemia					
	Severe		44 (55.7)	53 (60.9)	0.495
	Frequent		18 (22.8)	20 (23)	0.975
	Nocturnal		37 (46.8)	31 (35.6)	0.143
Previous ICU admission			35 (44.3)	36 (41.4)	0.704
Diabetes hospitalization			47 (59.5)	50 (57.5)	0.792
Family support			45 (57)	41 (47.1)	0.205
Physical activity			26 (32.9)	42 (48.3)	**0.044**
Glucose self-monitoring			74 (93.7)	82 (94.3)	0.875
Psychological assessment			49 (62)	40 (46)	**0.038**
Psychiatric medication			32 (40.5)	13 (14.9)	**<0.001**
Drugs					
	Alcohol		13 (43.3)	17 (56.7)	0.606
	Cigarette		7 (8.9)	9 (10.3)	0.746
	Cocaine		0 (0.0)	2 (2.3)	0.175
Suicidal			27 (34.2)	9 (10.3)	**<0.001**
SCI-R > 48			36 (45.6)	55 (63.2)	**0.022**
EAT ≥ 21			44 (55.7)	35 (40.2)	**0.046**
Depression			25 (31.6)	9 (10.3)	**<0.001**
Anxiety			47 (59.5)	20 (23)	**<0.001**

Data are presented as median (interquartile range), means ± standard deviation and number of patients (percentual).

1 Brazilian Problem Areas in Diabetes Scale.

2 Values ≥ 40 indicates a high level of emotional distress. Psychiatric medication means current treatment. Psychological assessment corresponds to assessment at any time in the past.

Treatment adherents totaled 91 (54.8%) using the SCI-R questionnaire. The group with adhesion showed better levels of glycated hemoglobin (8.4% [7.5%-9.1%] vs. 8.8% [8.0%-9.6%], p = 0.023), (68 mmol/mol [58-76] vs. 73 mmol/mol [64-81]), more frequent physical activity (50 [54.9%] vs. 18 [24%], p < 0.001), more self-blood glucose monitoring (90 [98.9%] vs. 66 [88%], p = 0.003) and lower frequency of psychiatric medication use (18 [19.8%] vs. 27 [36%], p = 0.019) in relation to patients poorly adherent. Smoking was more frequent in T1D patients with low adherence to treatment when compared with adherents (11 [14.7%] vs. 5 [5.5%], p = 0.046) (Supplement).

## DISCUSSION

Our study describes a cohort of T1D patients in two tertiary care centers for patients with diabetes in southern Brazil. Our findings show that depressed patients with high suffering related to diabetes had worse glycemic control, although we did not observe an association between psychiatric disorder and the presence of chronic complications of T1D.

In the present study, depressed patients are also more prone to severe hypoglycemia and a greater need for hospitalization due to the disease. Depressed patients were more prone to eating disorders. They also had less adherence to the proposed treatment and high levels of emotional distress related to T1D (B-PAID). In addition, they had a significant suicidal behavior or ideation, mainly in women.

Anxiety was related to sedentary behavior and low adherence to daily glycemic self-monitoring. It was more frequent in females. Those with anxiety disorder had statistically higher emotional distress related to diabetes (B-PAID), which perhaps explains the 72.2% of anxious patients with some thought of ideation and in some cases even attempted suicide. In addition, anxiety was strongly related to low adherence to diabetes treatment, although not significant, which can probably be related to our sample size.

Regarding to prevalence, we observed 20.5% and 40.4% of depressed and anxious patients, respectively. While Maia and cols. reported only 13.6% of depressed T1D patients, using the Hospital Anxiety and Depression Scale (HADS) tool for diagnosis (25) and data from the meta-analysis by Buchberger and cols. reported depression in 30% of children and adolescents with T1D (4). These disparities can be explained by different patient populations, however, all report significant numbers of these clinical conditions.

We found almost 55% of the screened sample with a positive result for some psychiatric disorder, of which 68% had already been evaluated by psychologists/psychiatrists at some point in their lives and only 27% were using pharmacological treatment. It is known that underdiagnosed and, consequently, undertreated patients worsen the prognosis of diabetes-related outcomes (6,14). In this context, psychological/psychiatric screening tools are essential for assess psychiatric pathologies. In our sample, the questionnaires detected about 1/3 of the patients with positive screening for psychiatric illness, without ever having been evaluated by a mental health professional.

More adherent patients had better glycemic control, while high levels of diabetes-related suffering were associated with an increased risk of depression and anxiety. In anxious patients, the prevalence of physical inactivity was higher than in controls. In the context of evaluating psychiatric comorbidities, sedentary patients with high levels of emotional distress seem to be more likely to develop such pathologies, even before they express symptoms of depression or anxiety. Therefore, the active search for such a condition should be encouraged and even should be part of the screening for chronic complications of diabetes.

According to the ADA, physical exercise is recommended for every T1D patient (26) and should be regularly stimulated. Our study demonstrated that the practice of regular physical activity was more frequent in the group without anxiety and was related to less psychological suffering related to diabetes and more adherence to the proposed clinical treatment (SCI-R).

The demands for self-care of T1D increase the risk of developing psychiatric disorders (4–6). A frequent problem in clinical practice is that mental health professionals are not always readily available to perform a comprehensive analysis of the patients’ psychological profile. In this scenario, screening questionnaires for depression, anxiety and eating disorders should be considered for use (26). We observed that almost half of the patients had positive screening for eating disorders, something that often neglected in routine medical care. Paradoxically, our patients with positive screening for eating disorder did not have a worsening of glycemic control, unlike all other psychiatric disorders assessed and several previous studies, which found worsening of glucose levels and an increased risk of hypoglycemia in patients with an eating disorder (27–29). As an example, Rydall and cols. found significantly higher HbA1c values (11.2% ± 1.2% or 99 ± 10 mmol/mol) in the group with severe eating disorder and T1D in relation to patients with diabetes without the disorder (8.7% ± 1.7% or 72 ± 5 mmol/mol) (30).

Regarding the limitations, we carried out a cross-sectional study, collecting some clinical data in medical records. Due to the design, we are limited in the definition of some vascular complications of diabetes, such as macrovascular disease and other comorbidities (hypertension, dyslipidemia and microvascular complications related to T1D). The diagnostics made by simple record in the medical record. This may certainly have underestimated the actual number of patients with vascular complications and the potential risk of inadequate data filling. However, since both clinics are university centers specialized in diabetes, the medical record information is reliable. Furthermore, we do not estimate the relevance of neuropathic pain in the psychological health of the participants. When using self-administered questionnaires that assess diabetes-related stress, we assumed the risk of misdiagnosing patients as depressed, which may have increased the frequency of depression, according to Roy and Lloyd (31). In addition, self-reported data can generate misleading results. Also, the lack of application of a more appropriate physical activity questionnaire underestimated our favorable finding regarding physical activity. We opted for the EAT-26 questionnaire because it was validated for the Brazilian population, even though it is not a specific tool for T1D patients, wich may have generated distortions in the results given the diabetes particularities and made it impossible to evaluate the diabulimia disorder when someone reduces or stops taking their insulin to lose weight (28).

The strengths of our study were the multicenter design, a broad evaluation of psychiatric disorders, the use of well-validated questionnaires and the finding of a huge number of patients with T1D have underdiagnosed and undertreated psychiatric disorders, which can impair glycemic control. Moreover, we found a high prevalence of eating disorders, psychiatric disorders and emotional distress related to type 1 diabetes. In particular, depression and elevated B-PAID were associated with worse glycemic control. Sedentary behavior and emotional distress or illness-related stress were associated with the presence of these pathologies. Therefore, medical care for psychiatric disorders should be encouraged by current guidelines and screening for these morbidities should be performed actively and early.
